# Molecular Origins of Thermoplastic Elasticity of Highly Branched Polyethylene as Revealed by Solid-State NMR Spectroscopy

**DOI:** 10.1021/acs.macromol.6c01367

**Published:** 2026-07-17

**Authors:** Bohao Peng, Keaton M. Turney, Walter G. Romano, James M. Eagan, Toshikazu Miyoshi

**Affiliations:** School of Polymer Science and Polymer Engineering, The University of Akron, Akron, Ohio 44325-3909, United States

## Abstract

Highly branched polyethylenes (HBPEs) synthesized via α-diimine nickel catalysis exhibit excellent thermoplastic elasticity due to the unique combination of long and short branching structures. It is, however, challenging to analyze the crystalline structure, crystallinity, and crystal size distribution of HBPEs due to their low crystallinity. Therefore, the relationship between the branching structure, solid structure, and mechanical properties has not been established. In this work, we provide a detailed structural characterization of various HBPEs by using solid-state ^13^C NMR spectroscopy combined with magnetic relaxation measurements. The combination of two ^13^C spin–lattice relaxation methods quantitatively analyzes the crystallinity, as well as size distributions of both the orthorhombic and monoclinic phases. By quantitatively comparing the detailed branching microstructures with crystallinity, it is established that HBPEs with long chain branching (LCB < 12b/1000C) and short-chain branching (SCB < 85 b/1000C) exhibit semicrystalline features. Furthermore, it was found that high fractions of small crystallites relative to the large ones, rather than crystallinity, lead to excellent strain recovery behavior. In conclusion, solid structures, including crystallinity, polymorph, and crystalline size distribution, govern the unique thermoplastic elasticity of HBPEs. This research provides guidance for designing branching microstructures for tunable solid structures and the mechanical properties of HBPEs.

## Graphical abstract

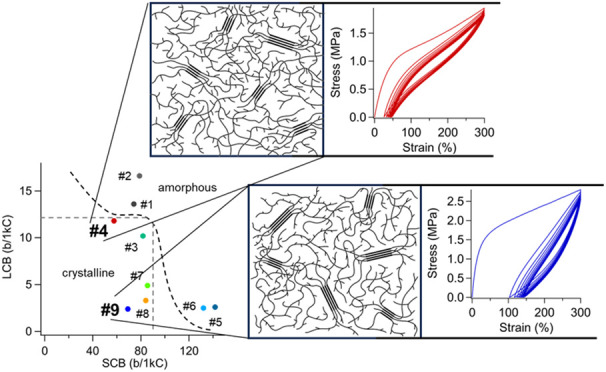

## 1 Introduction

Highly branched (HB) polyethylene (PE) with a branching number of up to over 100 branches per 1000 carbons (b/1kC) and variable branching microstructures have been successfully synthesized from a sole ethylene monomer since the discovery of the α-diimine nickel/palladium catalyst by Brookhart et al. in 1995.^1–3^ In the recent decade, it was found that HBPEs are excellent thermoplastic elastomers (TPEs) comparable to commercialized styrenic TPEs and polyolefin TPE,^4–11^ with a strain recovery of up to 91% after 10 cycles of stretching to 300% of the strain.^12^ The TPE property originates from the multiphase solid structure of the easily elongated soft domain physically cross-linked by the hard domain,^13–18^ where, in the case of HBPE, the former and latter refer to the flexible amorphous phase and rigid crystalline phase, respectively.^4,6^ Besides elasticity, it is well known that the mechanical properties of PEs, such as stress at break and strain at break, are influenced by various parameters, including the molecular weight, branching structure, crystallinity, and crystalline size, *etc*.^6,19^ The highly branched nature results in low crystallinity or a completely amorphous state, which is mainly confirmed by differential scanning calorimetry (DSC)^3,7–10,20^ and X-ray diffraction (XRD).^21,22^ With decreasing crystal size, both the melting^3,23,24^ and diffraction peaks^22,25^ broaden. It is expected that the DSC and XRD peaks will diminish at a certain level of low crystallinity. Therefore, the role of the crystalline structure in thermoplastic elasticity, along with the correlation between the branching microstructure and solid structure, has not been elucidated.^6,26^

Alternative methods to characterize a minor crystalline structure include spectroscopic techniques such as Raman, FT-IR, and solid-state NMR (ssNMR) spectroscopy.^19,27,28^ Among these, ssNMR spectroscopy has been applied to conventional PE systems of high crystallinity (>20%),^29–31^ which can identify crystalline polymorphs of the orthorhombic and monoclinic phases in addition to the amorphous signal at different chemical shifts.^25,32–36^ Furthermore, magnetic relaxations reflect chain mobility, which is distinct between the crystalline and amorphous phases.^37–40^ Therefore, a combination of magnetic relaxations with high-resolution ^13^C ssNMR spectroscopy is theoretically capable of highlighting a very small crystalline component at a ca. 1% level. In the early days, Alexon et al. reported the relationship between the ^13^C spin–lattice relaxation time (*T*_1C_) obtained by ssNMR and crystalline sizes analyzed by the Raman-active longitudinal acoustic vibrational mode.^37^ Therefore, the magnetic relaxation methods of ssNMR are very powerful to understand the crystalline structure, crystalline size, and crystallinity of HBPEs.

In this study, we aim to explore the microstructure-solid structure–property relationships of HBPEs. We use nine HBPE samples with a branching number of 70–140 b/kC, which we very recently synthesized using three different α-diimine nickel catalysts,^41,42^ in which the one with the best elasticity shows a strain recovery of 84% after 10 cycles of stretching to 300% of the strain. First, we review the detailed branching microstructure of the nine HBPE samples.^41^ Second, we investigate their thermal properties and solid structures using DSC, XRD, and ssNMR. Only ssNMR spectroscopy selectively observes the buried crystalline structure at the 1% level by filtering out the major noncrystalline component.^31^ The combination of the two relaxation methods allows us to identify the two crystalline phases, orthorhombic and monoclinic, and determine the crystalline size distribution and crystallinity (1.3–6.2%) of semicrystalline HBPEs. Third, the mechanical properties of the seven HBPE samples are characterized through tensile tests and stress-recovery hysteresis experiments. Finally, the relationship between the branching microstructures, solid crystalline structures, and mechanical properties is discussed. We rationalize how three crystalline parameters, namely crystallinity, crystalline size distribution, and polymorph, could affect the mechanical properties of HBPEs. Therefore, this research correlates both the microstructure and mechanical properties with their crystalline structures.

## 2 Experimental Section

The synthesis protocol, molecular weight characterizations, and ^13^C solution-state NMR experiments of nine HBPE samples have been previously reported.^41^ In the current work, samples characterized in the solid state are prepared in the same way as for mechanical testing. Samples were compression-molded into tensile dogbone specimens at 180 °C for 5 min, followed by air cooling.

An HDPE sample with a weight-average molecular weight of 782 K g/mol and a dispersity of 4.1 was included as a reference. HDPE was synthesized using 9 mg of the Rac-di­methyl­silyl­bis­(1-indenyl)­zirconium dichloride (SiZr) catalyst, 1.04 mg of polymethylaluminoxane (PMAO) cocatalyst, 50 mL of dry toluene, 1.05g of ethylene gas, and a reaction time of 3 h. The detailed synthesis procedures and materials are provided in Supporting Information (SI). The HDPE sample in a sealed glass was heated in an oil bath at 210 °C for 15 min and then quickly transferred into the oil bath at 110 °C and crystallized for 30 min.

DSC experiments were conducted using a TA Discovery DSC 250 with heating and cooling rates of 10 °C/min in a nitrogen gas atmosphere. A heat–cool–heat progress was conducted. XRD measurements were performed using a Rigaku Rapid II Diffractometer at ambient temperature. The X-ray source was Cu Kα, where the tube voltage was set to 40 kV, and the tube current was set to 30 mA. A 0.8 mm collimator was used during the test. Each profile was accumulated with an exposure time of 15 min. A linear background was subtracted.

The ssNMR experiments were carried out on a BRUKER AVANCE 300 spectrometer, equipped with a 4 mm double-resonance probe. The carrier frequencies for ^1^H and ^13^C were 300.1 and 75.5 MHz, respectively. All solid-state NMR experiments in this study were conducted at a magic angle spinning (MAS) frequency of 4000 Hz at ambient temperature. The 90° pulses for ^1^H and ^13^C were adjusted to 3.3 and 4.5 μs, respectively. High-power ^1^H two-pulse phase modulation (TPPM) decoupling with a field strength, γB_1_/2π, of 75.8 kHz was used during the ^13^C acquisition time. Cross-polarization (CP)­MAS experiments were conducted with a CP time of 1 ms and a recycle delay of 2 s. Torchia pulse sequence,^43^ denoted as CP-*T*_1C_ experiments, was used to determine the ^13^C spin–lattice relaxation time (*T*_1C_), where the number of scans is in the range of 32 to 4096. The saturation recovery pulse sequence,^40^ denoted as SR-*T*_1C_ experiments, was used to determine the crystallinity of the samples, where the number of scans for the HDPE sample was in the range of 16 to 1024. The number of pulses in the saturation pulse train and the repetition time were set to 20 and 20 μs, respectively. For each experiment above, around 50 mg of the sample was packed into the 4 mm rotor for characterization. The ^13^C chemical shift was externally referenced to the methine peak of adamantane at 29.46 ppm.

The mechanical tests were performed using a Universal Test Frame Instron 5567 and a 100 N load cell at ambient temperature. All samples were prepared by heat pressing at 180 °C for 5 min into tensile specimens with dimensions of 10 mm in length, around 3 mm wide, and 1 mm thick. Before testing, the width and thickness of each sample are measured with a caliper and input into the equipment to ensure that an accurate pressure is recorded. The tensile rate for all tests was 10 mm/min.

## 3 Results

### 3.1 Branching Microstructure

In our previous work, we investigated the branching number, pattern, and distribution of nine HBPEs synthesized using three α-diimine nickel catalysts with different ligand structures, namely Brookhart catalyst (BH), methyl-sandwich catalyst (MS), and cross-linked catalyst (XL).^41^ Since the branching microstructures significantly affect the solid structure as well as mechanical properties, we reproduce the detailed microstructures of nine HBPEs in Table 1. These HBPEs show different characteristic features in the branching microstructures, such as the branching number and branching pattern. The important structural differences are highlighted: Entries 4 and 9 have low branching numbers of around 70 b/1kC, which is the lowest number among the nine samples. Entries 1, 2, 3, 7, and 8 have medium branching, with around 90 b/1kC. Entries 5 and 6 possess the highest branching, with around 140 b/1kC. In terms of branching pattern, BH entries have the highest LCB ratio (10–17 b/1kC), followed by XL entries (2–5 b/1kC) and MS entries (2–3 b/1kC). MS entries such as entry 6 contain 124 methyl branches/1kC, which constitutes 96% of the total branching number. These microstructures are known to influence the crystallinity, thermal, and mechanical properties.^19^ However, the impact of these branches on the crystal structure as well as the elastomeric behavior of HBPEs is not known.

**Table 1:** Molecular and Thermal Properties, and the Branching Structures of All HBPE Enteries

		*M*_n_ ^a^	*Đ* ^a^	*T*_g_ ^b^	*T*_c_ ^b^	*T*_m_ ^b^	*N*_b_ ^c^	branching pattern [b/1kC]^c^						
entry no.	cat	[kg/mol]	[*M*_w_/*M_n_*]	[°C]	[°C]	[°C]	[b/1kC]	Me	Et	Pr	Bu	Am	He	LCB
1	BH	163	2.3	–61			87.74	59.9	6.3	2.7	3.1	2.1		13.6
2	BH	67	2.6	–65			95.39	61.5	9.2	2.7	4.3	1.1		16.6
3	BH	241	2.8	–61			92.07	68.7	5.6	2.9	2.9	1.7		10.2
4^d^	BH	100	2.9	–52	15		69.57	39.7	2.4	1.7	1.7	1.6	10.6	11.8
5	MS	73	3.1	–64			144.42	134.1	2.9	1.8	1.6	1.4		2.6
6	MS	64	4.3	–61			134.38	124.3	2.7	1.7	1.9	1.4		2.5
7	XL	68	3.3	–61	65		90.35	72.6	7.7	2.5	2.0	0.8		4.9
8	XL	66	3.4	–61	62		87.40	74.1	5.2	2.2	1.7	0.9		3.3
9	XL	83	3.8	–53	87	112	71.60	61.9	3.6	1.9	1.2	0.6		2.4

a *M_n_* and *Đ* were determined by gel permeation chromatography (GPC) in 1,2,4-trichlorobenzene at 140 °C using a polystyrene standard (*Đ* < 1.1) for calibration.

b Glass transition, crystallization, and melting temperature were determined by DSC at 10 °C/min.

c Branching number (*N*_b_ is the total number of branches per 1000 carbons) and the pattern (the number of different types of branches per 1000 carbons) were determined by solution-state ^13^C NMR spectroscopy at 125 °C.

d Octene commoner was incorporated into the polymerization process.

### 3.2 Crystalline Structure

DSC experiments were conducted on all HBPE samples at heating and cooling rates of 10 °C/min from −90 to 180 °C, as shown in Figure S1. The results are summarized in Table 1, where the glass transition temperature (*T*_g_) is observed for each entry in the range of −52 to −65 °C. Among the nine samples, entries 4, 7, 8, and 9 showed a tiny crystallization peak during cooling, whose temperatures (*T*_c_) are 15, 65, 62, and 87 °C, respectively. We present the second DSC heating curves for two samples, entries 8 (orange) and 9 (blue), along with an HDPE sample (black) in Figure 1. Compared to the melting peak of HDPE at 136.5 °C, entry 9 exhibits a very weak melting peak at 112 °C, which includes a long tail on the low-temperature side. It is challenging to unambiguously identify the onset of the melting peak. Different onset temperatures lead to different enthalpies of 54.0, 16.5, 10.7, and 5.9 J/g for a, b, c, and d, respectively, and the corresponding crystallinities of 18.4, 5.6, 3.4, and 2.0% for a, b, c, and d, respectively, using 293 J/g as Δ*H*_f,100%_.^19^ Such broadening behavior makes it difficult to determine the crystallinity of HBPEs. Furthermore, the mechanical properties were determined at ambient temperature, where part of the crystals might melt. Among the nine samples, the other 8 samples, including entry 8 (Figure 1), do not show a melting peak upon heating. Therefore, it is not suitable to estimate the crystallinity of HBPEs by using DSC, which sweeps a certain temperature range.

**Figure 1:**
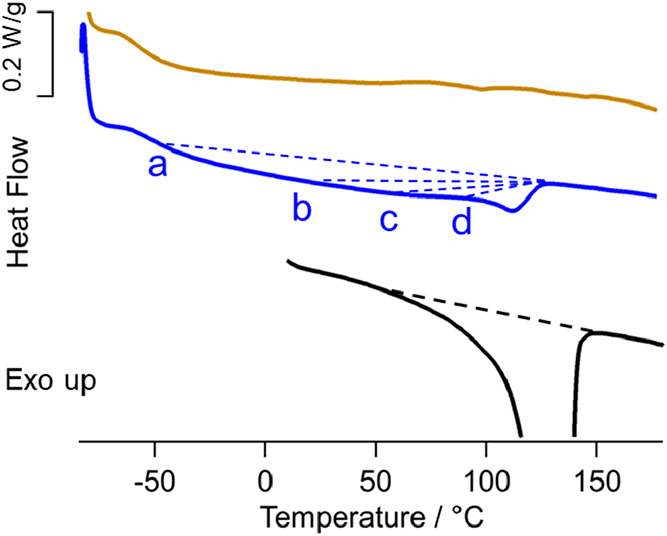
Second heating curves of HBPE entries 8 (orange), 9 (blue), and an HDPE sample (black).

Figure 2 shows the ^13^C solution-state (blue) and ssNMR spectra of entry 7. In the latter, two methods were used: saturation recovery with a recovery time of 200 s (black), which enables us to fully excite all the signals of HBPE. Alternatively, the cross-polymerization magic angle spinning (CPMAS) experiment (red) highlights the rigid components (i.e., crystalline signals). Compared to the solution-state spectrum, the observed peaks in the solid-state spectra shift downfield by 0.4–0.8 ppm. The noncrystalline methylene backbone peak of HBPE appears at around 30.8 ppm, and signature peaks associated with the branching structures, such as methyl carbon (1B_1_), methine carbon (brB_1_), and α-carbon (αB_1_) of methyl branches at 20.4, 33.4, and 38.0 ppm, respectively, were observed in both CPMAS and saturation recovery spectra.^41^ On the other hand, 1B_2_, 1B_n_, and 2B_n_ signals were not observed in the former. This indicates that these branching structures possess fast molecular dynamics, which results in lower CP efficiency at ambient temperature.

**Figure 2:**
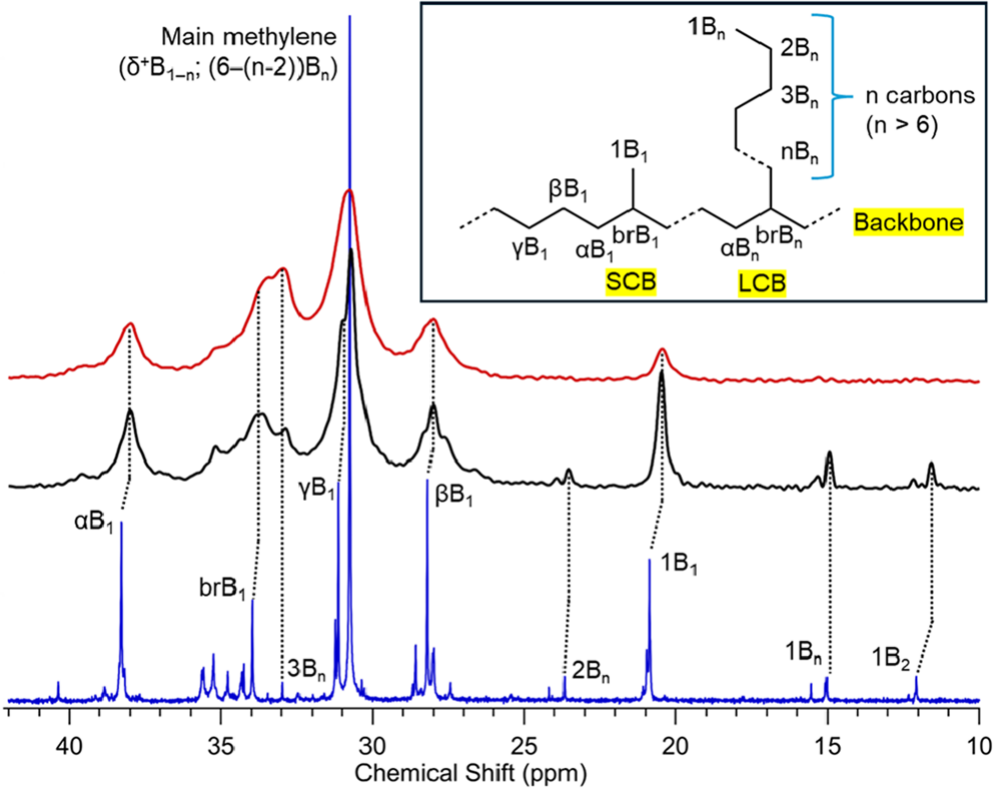
^13^C CPMAS (red) and SR-*T*_1C_ with a recovery time of 200 s (black) solid-state NMR spectra, as well as the ^13^C solution-state NMR spectrum (blue) of entry 7. The characteristic branching structure is denoted by “xB_n_”, as illustrated in the top right. The full peak assignments in the solution-state ^13^C spectrum are presented in previous work.^41^

Figure 3a,b depicts the XRD patterns and CPMAS NMR spectra, respectively, of all the entries. The former are dominated by a broad amorphous halo center at around *2θ* = 19.5°. For entries 3 and 7–9, two weak crystalline peaks appear at *2θ =* 21.3 and 23.4°, which are characteristic (110) and (200) peaks, respectively, of the orthorhombic cell of PE.^28^ According to the literature, the PE monoclinic phase (001), (200), and (−210) planes show diffraction peaks at 19.45, 23.17, and 25.11°, respectively.^36^ However, such peaks were not observed in any of the entries.

**Figure 3:**
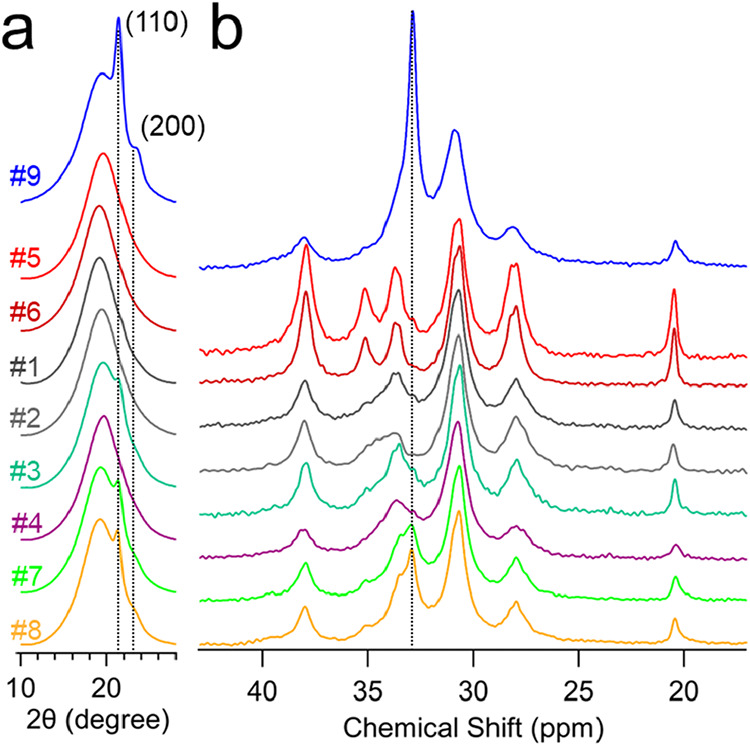
(a) 1D XRD patterns and (b) ^13^C CPMAS solid-state NMR spectra of all HBPE entries used in this work. The crystalline peaks are marked with dashed lines.

In the CPMAS NMR spectra, the crystalline signals in the orthorhombic and monoclinic phases appear at around 32.9 and 33.9 ppm, respectively.^34,36^ Notably, entries 3, 7, 8, and 9 have a relatively higher intensity at 32.9 ppm than the others. However, the noncrystalline signal, such as the third methylene on an LCB (3B_n_), overlaps with the crystalline peak, as represented in Figure 2. The PE chains in the crystalline and noncrystalline regions possess largely different chain mobilities, which are reflected in differences in magnetic relaxations, such as the spin–lattice relaxation time (*T*_1_) and spin–spin relaxation (*T*_2_).^30^ Therefore, the relaxation-filtered ^13^C NMR spectrum can highlight the buried crystalline signals at ca. the 1% level.

Figure 4a–d depicts the ^13^C *T*_1_-dependent CPMAS NMR spectra for the HDPE sample and HBPE entries 1, 8, and 4, respectively. In HDPE, the amorphous peak at 31.0 ppm decays within 1 s, whereas the orthorhombic peak at 32.9 ppm does not fully decay even at a relaxation time of 2500 s. The rapid decay of the PE amorphous chains is due to a rapid conformational transition between the *trans* and *gauche* chains, whose frequency is close to the ^13^C Larmor frequency of 75 MHz at ambient temperature.^40,44–46^ Meanwhile, the PE crystalline chains only conduct 180° chain flips with a frequency of 10 Hz at the same temperature,^47^ which cannot effectively shorten *T*_1C_. The HBPE entries are categorized into two groups based on the presence or absence of the crystalline peak at 32.9 and 33.9 ppm at a delay time of 2s. Note that entry 1 gives a very tiny peak at 32.9 ppm at 2s. However, the alternative method of saturation recovery fails to recognize the crystalline signal. Therefore, entry 1 is treated as a noncrystalline one. In summary, entries 1, 2, 5, and 6 are noncrystalline, whereas the remaining entries 3, 4, 7, 8, and 9 are semicrystalline. For semicrystalline HBPE entries, as illustrated in Figure 4c for entry 8, the relaxation behaviors are similar to those of the noncrystalline ones, except for the semicrystalline peak at 32.9 ppm, where rapid and slow components were observed. Furthermore, two crystalline peaks at 32.9 and 33.9 ppm are observed in the NMR spectra of entry 4 (Figure 4d) and entry 3. The former and latter peaks are assigned to the orthorhombic and monoclinic phases, respectively.^25,34,36^

**Figure 4:**
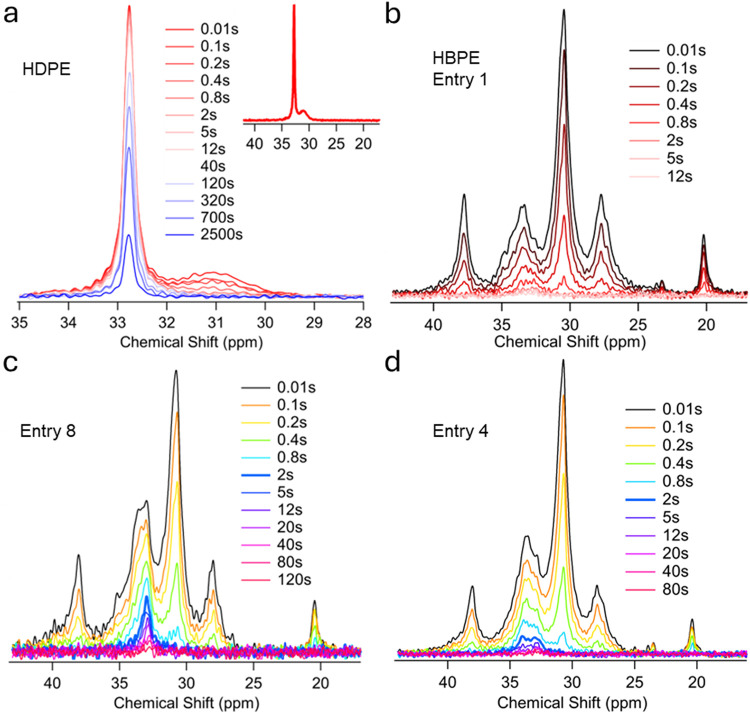
CP-*T*_1C_ spectra of (a) an HDPE sample; HBPE entries 1 (b), 8 (c), and 4 (d) under variable relaxation delay times. The spectrum at a delay time of 2s is highlighted in bold blue color in (c) and (d), where only crystalline peaks are observed.

To determine the crystalline *T*_1C_ values, the semicrystalline peaks were deconvoluted through Lorentzian peak fitting on each sliced spectrum, as shown in Figure 5. Then, the relaxation of the semicrystalline peak at 32.9 ppm was deconvoluted into three components, which are assigned to amorphous (“a”), short-*T*_1C_ crystalline (“short”), and long-*T*_1C_ crystalline (“long”), components, respectively: *A*(*t*) = *A*_0_·[*f*_a_ exp­(−*t*/*T*_1C,a_) + *f*_s_ exp­(−*t*/*T*_1C,short_) + *f*_l_ exp­(−*t*/*T*_1C,long_)], where *f*_a_ + *f*_s_ + *f*_l_ = 1. *A*(*t*) is the peak area at a delay time *t*, *A*_0_ is the fitted parameter representing the initial area at 0 s, and *f* corresponds to the ratio of each component. On the other hand, the relaxation of the semicrystalline peak at 33.9 ppm (monoclinic phase) of entries 3 and 4 shows a double exponential behavior and is fitted as *A*(*t*) = *A*_0_·[*f*_a_ exp­(−*t*/*T*_1c,a_) + *f*_short_ exp­(−*t*/*T*_1C,short_)]. In summary, the crystalline *T*_1C_ values can be well fit into the integration area analysis, and the amorphous *T*_1C_ and its ratio around the crystalline peaks are also determined.

**Figure 5:**
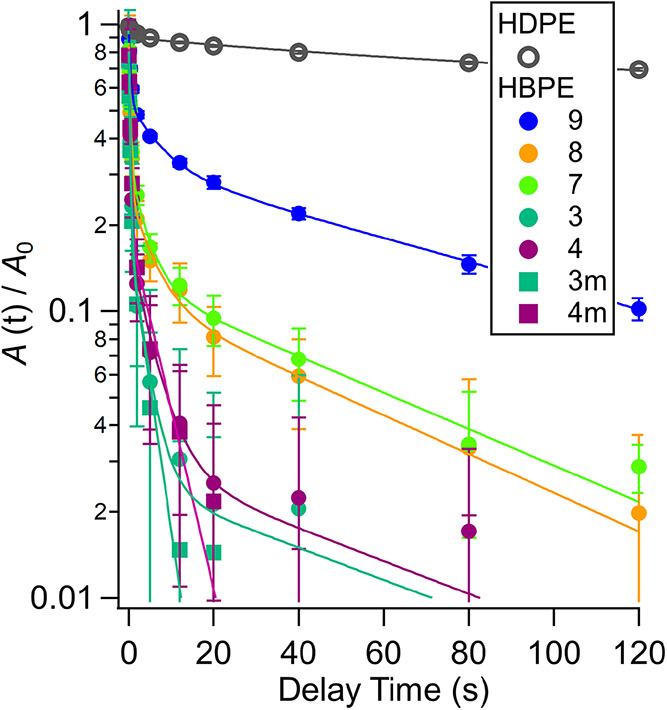
CP-*T*_1C_ relaxation behaviors of HDPE and HBPE entries 9, 8, 7, 3, and 4. The full relaxation curve of the HDPE sample is shown in Figure S3a.

The HDPE sample shows *T*_1C_ = 1876 and 103.9 s, which are close to the *T*_1C_ values of the linear PEs in the literature^37,48^ (e.g., sample c in ref 38, 1695 s for *T*_1C,l_). Except for HBPE entry 9, which has *T*_1C,l_ of 104.5 s, all other HBPE entries show *T*_1C,l_ of around 70 s, with *T*_1C,s_ ranging from 3.3 s (entry 3) to 7.2 s (entry 9). These crystalline relaxation values are much smaller than those of the HDPE sample in this work and LDPE in the literature^35,37,38,49,50^ and are close to PE with a high ratio of the olefin comonomer (∼10 mol %) in several studies^23,25,50–52^ (e.g., ethylene-butene copolymer in ref 45, *T*_1C_ = 140 s, 14 s). The *T*_1C_ values of the monoclinic crystalline peaks of entries 3 and 4 are 4.3 and 6.9 s, respectively, which are much shorter than those of the orthorhombic crystalline peak. This tendency is consistent with the results of the literature.^25^ The amorphous *T*_1C_ at the crystalline peak position (*T*_1c,a_) of the HDPE sample is 1.1 s, while those of the HBPEs are around 0.3–0.4 s. This difference indicates the contribution of the branching signals at the crystalline peak position, including 3B_n_ on LCB and others, which HBPE has and HDPE does not. As the LCB number increases, the amorphous *T*_1C_ ratio (*f*_a_) increases, such as in entry 9, which has the lowest *f*_a_ of 46%, entries 7 and 8 have *f*_a_ of around 70%, and entries 3 and 4 have *f*_a_ of over 80%. Meanwhile, cross-polarization (CP) techniques also modulate the crystalline ^13^C signal intensity depending on mobilities.^34^ Therefore, the *f*_a_ determined from the CP-based relaxation analysis cannot be directly related to the crystallinity.

To quantitatively determine the crystallinity of the HBPEs, the saturation recovery (SR) method was employed to measure the proper intensity ratio of the crystalline signal to the noncrystalline signal.^40^ Figure 6a presents the ^13^C saturation recovery sliced spectra of entry 9 as a function of the recovery time. The signal intensity increases with increasing recovery time. Only the crystalline peak at 32.9 ppm continues to develop at recovery times longer than 1 s, while the other peaks reached equilibrium intensities within 1 s. The fully relaxed ^13^C spectrum is the full polarization of the entire signals, and therefore, quantitatively provides the signal intensity of both the noncrystalline and crystalline regions.

**Figure 6:**
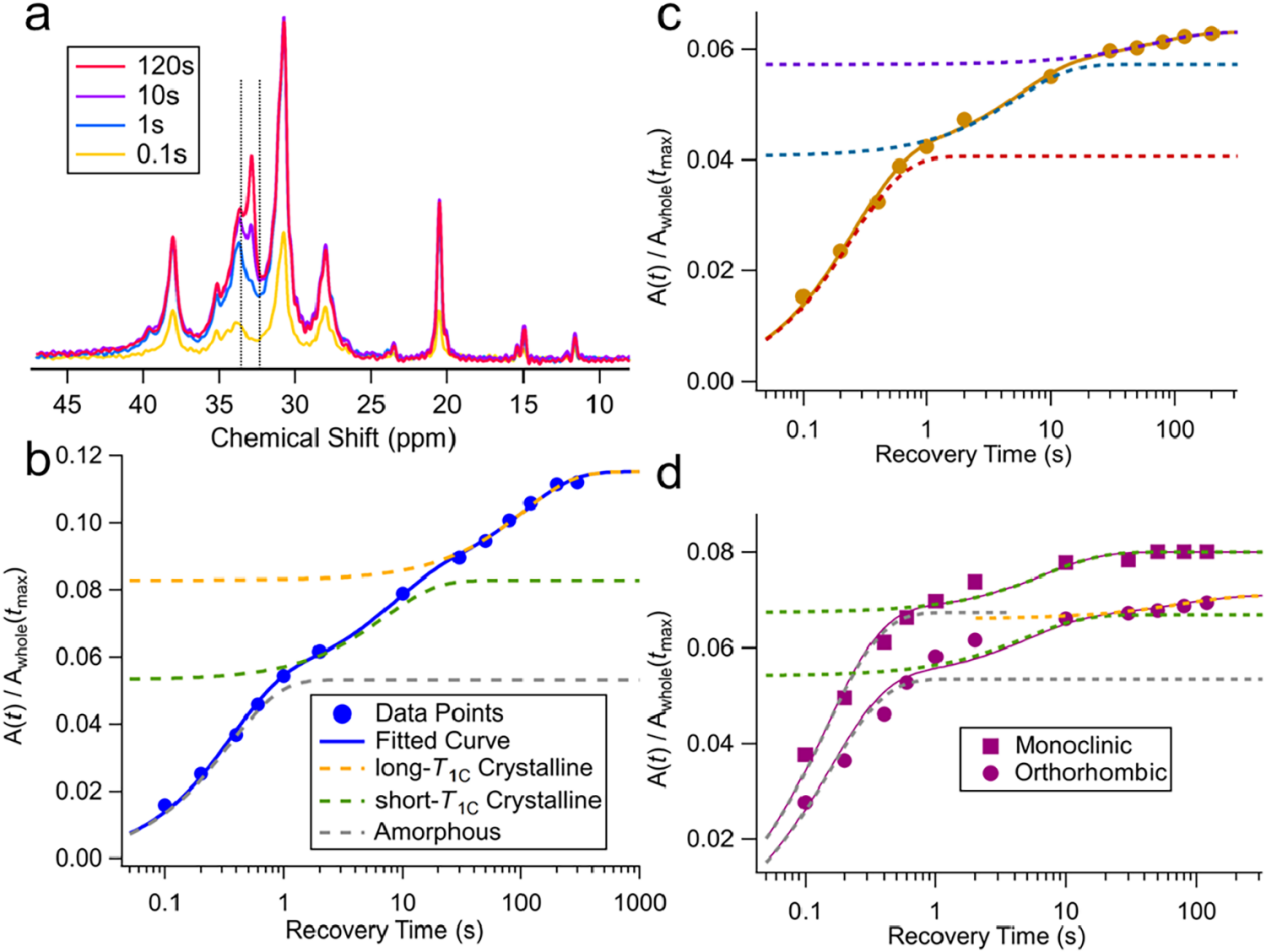
(a) SR-*T*_1C_ spectra of HBPE entry 9 under variable recovery times. Two dashed lines are drawn to denote the peak integration region used for further analyses. (b) Signal recovery in the SR-*T*_1C_ experiment in the spectral region from 32.4 to 33.4 ppm for entry 9 and its three-component fitted curve. Three additional illustrative dashed lines are drawn to indicate the signal recovery from each component. (c) Signal recovery in the SR-*T*_1C_ experiments of entry 8 and (d) entry 4. Signal recovery in the SR-*T*_1C_ experiments of the HDPE sample and HBPE entries 7 and 3 are shown in Figures S3b, S4, and S5, respectively. The best-fitting parameters of these fitted curves are presented in Table S2.

Figure 6b shows the SR signal recovery, *A*(*t*)/*A*_whole_, of entry 9, where *A*(*t*) and *A*_whole_ correspond to the peak area in the region from 32.4 to 33.4 ppm and the whole signal of entry 9, respectively. Therefore, the *y-*axis provides the crystallinity. From the plot, we can see a rapid signal buildup before the recovery time of 1 s, which mainly comes from the noncrystalline branching signals. After 1 s, the signal further increases due to the recovery signal from the crystalline region. Since the CP-*T*_1C_ measurement revealed that the crystalline signal consists of two components, short-*T*_1C_ of 7.1 s and long-*T*_1C_ of 104.5 s, in addition to the noncrystalline branching signal, a triple relaxation model was used to fit the buildup data:

$$A\left(t\right)=A\left(t_{\max}\right)\left[f_{a}\left(1-\exp\left(-\frac{t}{T_{1C,a}}\right)\right)+f_{s}\left(1-\exp\left(-\frac{t}{T_{1C,\mathrm{sh}ort}}\right)\right)+f_{1}\left(1-\exp\left(-\frac{t}{T_{1C,\mathrm{lo}\mathrm{ng}}}\right)\right)\right]$$

where *f*_a_, *f*_s_, and *f*_l_ correspond to the ratios of the noncrystalline branching signals, short-*T*_1C_ crystalline and long-*T*_1C_ crystalline signals, respectively, and *f*_a_ + *f*_s_ + *f*_l_ = 1. *T*_1C,a_ is *T*_1C_ of the noncrystalline signal, and *T*_1C,l_ and *T*_1C,s_ values obtained from the CP-*T*_1C_ experiments were applied for the analysis because in the SR experiment, the noncrystalline components that do not contribute to the CP signal dominate the whole relaxation process, and crystalline relaxation does not significantly contribute to the whole signal recovery (see also Figure S6). In Figure 6b, the three dashed curves correspond to the single-exponential curves of the amorphous, long-*T*_1C_, and short-*T*_1C_ crystalline components, respectively, which show that the signal buildup of the short-*T*_1C_ crystalline component mainly occurs at the recovery time between 1 and 10 s, and that of the long-*T*_1C_ crystalline component apparently appears above 10 s. By multiplying *f*_l_ and *f*_s_ with the ratio of the selected region to the whole spectrum (11–41 ppm) at the maximum recovery time (*A*(*t*_max_)/*A*_whole_(*t*_max_)), the crystallinity (χ_total_) of entry 9 is determined to be 6.2%, in which the long-*T*_1C_ crystallinity (χ_l_) is 3.2% and the short-*T*_1C_ crystallinity (χ_s_) is 3.0%. Meanwhile, as we used the ratio of the selected integration area at a recovery time *t* (*A*(*t*)) to the area of the whole spectrum (11–41 ppm) at the maximum recovery time (*A*_whole_(*t*_max_)) as the left axis, the crystallinity can be directly observed from the graph through the vertical distance between the dashed lines.

In the same way, the SR signal recovery analysis is obtained for entry 8, as shown in Figure 6c. At first glance, the signal recovery above 1 s is much smaller than that of entry 9, which implies that χ_total_ is lower than 6.2%. The signal recovery between 1 and 10 s is higher than that above 10 s, corresponding to a higher χ_s_ than χ_l_. Through crystallinity calculation, entry 8 has a χ_total_ of 2.3%, composed of χ_s_ of 1.7% and χ_l_ of 0.6%. Entry 7 has a χ value similar to entry 8, as shown in Figure S4 and Table 2. The SR signal recovery analysis of entry 4 is shown in Figure 6d, where the regions from 32.4 to 33.4 ppm and from 33.4 to 34.4 ppm are used to analyze the orthorhombic crystalline and monoclinic crystalline phases, respectively. As a result, the orthorhombic (χ_o_) and monoclinic crystallinities (χ_m_) of entry 4 are determined to be 1.9 and 1.3%, respectively. The monoclinic phase can be represented by only one short-*T*_1C_ component. Therefore, the crystallinity of entry 4 can also be interpreted as χ_s_ = 2.7% and χ_l_ = 0.5%. Entry 3 also has a monoclinic phase but shows a lower χ than that of entry 4 in each component, as shown in Figure S5 and Table 2. In summary, entry 9 has the highest χ_total_ of 6.2%, while the χ_total_ of the other entries ranges from 1.3% (entry 4) to 3.2% (entry 3), in which χ_o_ ranges from 0.8% (entry 3) to 2.4% (entry 7) and χ_m_ ranges from 0.5% (entry 3) to 1.3% (entry 4). The long- or short-*T*_1C_ crystalline components have different relative ratios among the samples, where χ_s_/χ_l_ ranges from 0.94 (entry 9) to 5.56 (entry 4). This ratio indicates the size distribution of the crystals, which will be elaborated on later.

**Table 2:** CP-*T*_1C_ Values and Ratios, and the Crystallinity of Each Entry

entry	crystalline phase^a^	*T*_1C,long_ ^b^/s	*f*_l_ ^c^/%	*T*_1C,short_ ^b^/s	*T*_1C,a_ ^c^/s	*f*_a_ ^c^/%	χ_l_ ^d^/%	χ_s_ ^d^/%
1	^e^	^e^	^e^	5.75 ± 2.21	0.36 ± 0.03	86	0	N/A^f^
2	^e^	^e^	^e^	^e^	0.24 ± 0.02	100	0	0
3	O	77.5 ± 19.1	2.5	3.30 ± 1.27	0.30 ± 0.01	80	0.4	0.4
	M	N/A	N/A	4.30 ± 0.64	0.28 ± 0.01	82	0	0.5
4	O	76.1 ± 9.0	3	5.00 ± 0.39	0.33 ± 0.01	82	0.5	1.4
	M	N/A	N/A	6.89 ± 0.84	0.34 ± 0.02	80	0	1.3
5	^e^	^e^	^e^	^e^	0.35 ± 0.04	100	0	0
6	^e^	^e^	^e^	^e^	0.30 ± 0.01	100	0	0
8	O	64.3 ± 13.6	11	5.29 ± 2.27	0.36 ± 0.02	73	0.6	1.7
7	O	68.4 ± 4.9	12	4.00 ± 0.59	0.34 ± 0.01	66	0.9	1.5
9	O	104.5 ± 6.8	32	7.14 ± 1.37	0.41 ± 0.02	46	3.2	3.0
HDPE	O	1876 ± 77	65	103.9 ± 14.0	1.14 ± 0.22	10	33.8	17.2

a Crystalline phase, where “O” denotes the orthorhombic crystalline phase and “M” denotes the monoclinic crystalline phase.

b Determined by deconvoluted peak area analysis from the CP-*T*_1C_ experiments (Figure 5).

c Determined by curve fitting in the peak integration area analysis (Figure 4).

d *T*_1C_ results from the former are applied in saturation recovery (SR) experiments to determine the long-*T*_1C_ crystallinity (χ_l_) and short-*T*_1C_ crystallinity (χ_s_) (see Figure 6).

e Not applicable to CP studies, and these samples were considered entirely amorphous.

f Below the analysis limit of the crystallinity of the SR experiments.

### 3.3 Mechanical Properties

Figure 7a shows the stress–strain curves for the HBPEs studied in this work. All semicrystalline and two amorphous entries (1 and 2) are chosen to study their stress–strain behaviors. Figure 7a and Table 3 show the stress–strain curves and characteristic mechanical properties, respectively, for the seven HBPE samples. The toughness (*U*) of the semicrystalline entries is much higher than that of the amorphous entries. The stress-at-break (σ) ranges from 0.45 MPa (entry 1) to 4.24 MPa (entry 9), and the strain-at-break (ε) ranges from 664% (entry 4) to 5256% (entry 3). The tested entries are roughly divided into four categories. Entries 1 and 2 are classified as category I, which are noncrystalline polymers showing small σ values (0.45–0.53 MPa) and small *U* (∼6 J·m^–3^). The semicrystalline entries are classified as categories II (entry 3), III (entries 7, 8), and IV (entries 4 and 9), respectively. Categories II and III contribute to a higher modulus than category I due to the presence of a crystallinity lower than 3.0%. The former corresponds to a high *U* (57.72 J·m^–3^) with superstretching behavior (ε = 5256%). The latter represents intermediate *U* (16–25 J·m^–3^) with moderate *ε* (1556%–2420%). Category IV represents a higher Young’s modulus (*E*) (3.25 and 8.47 MPa) and higher σ (3.61 and 4.24 MPa) than those of categories II and III. Furthermore, the strain recovery behaviors of the semicrystalline HBPEs were investigated, as shown in Figures 7b–d and S7 and S8. Interestingly, the strain recovery ratios of entries 3, 7, 8, and 9 are very similar in the range of 52–62%, whereas only entry 4 gives a ratio of 84% after 10 cycles.

**Figure 7:**
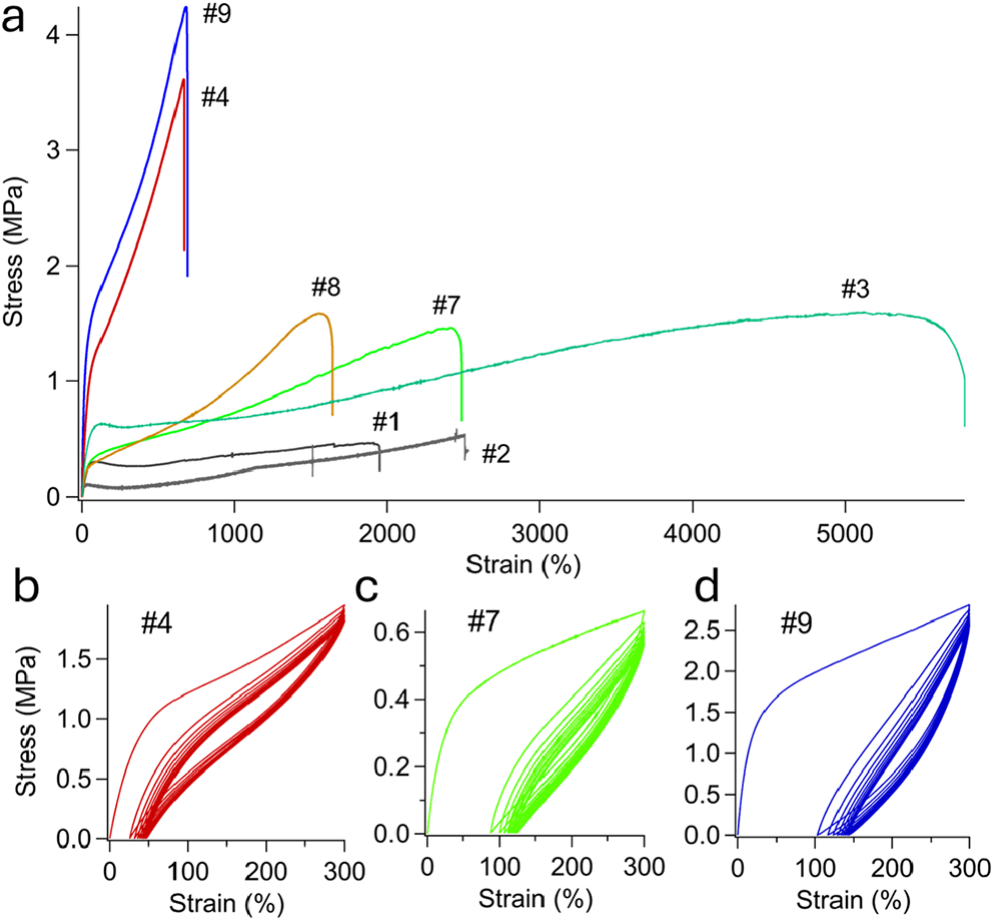
(a) Strain–stress curves from regular tensile testing for entries 1–4 and 7–9; stress–strain curves from hysteresis experiments with ten cycles up to 300% strain for (b) entry 4, (c) entry 7, and (d) entry 9. The hysteresis experiments for entries 3 and 8 are shown in Figures S7 and S8, respectively.

**Table 3:** Mechanical Properties of HBPE Entries

entry	σ^a^ (MPa)	ε^a^ (%)	*E* ^b^ (MPa)	*U* ^c^ (J·m^–3^)	ε_300%_10th^d^ (%)	ε_300%_^first^ (%)
1	0.45	1940	0.77	6.64		
2	0.53	2510	1.25	6.89		
3	1.59	5256	1.76	57.72	62	72
4	3.61	664	3.25	14.14	84	91
7	1.46	2420	1.64	24.89	58	70
8	1.58	1556	1.5	16.37	54	67
9	4.24	680	8.47	18.11	52	65

a Strain at break, ε, and stress at break, σ, were determined at fracture using a uniaxial tensile test.

b Young’s modulus, *E*, is the initial slope of the stress vs strain curve in the linear region (0 < ε < 5%).

c Toughness, *U*, was determined by integrating the tensile curve from zero strain to the strain at break.

d Recovered strain (RS) determined by a 300% strain step cycle test using the equation 100 (ε_a_–ε_r_)/ ε_a_, where ε_a_ is the applied strain, and *ε*_r_ is the strain at zero load after the 10th cycle.

## 4 Discussion

### 4.1 Detection Limit of Small Crystals

Different characterization techniques, including DSC, XRD, and ssNMR, are applied in this work to characterize the crystal-related solid structures of HBPEs. Through ssNMR, it was observed that entries 3, 4, 7, 8, and 9 are semicrystalline with a crystallinity in the range of 1.3–6.2%. All of them show an orthorhombic crystalline phase, while entries 3 and 4 show an additional monoclinic phase. The crystal size distribution (Table 2) is also obtained by *T*_1C_ experiments. Previously, Axelson et al. have reported that the crystalline *T*_1C_ value obtained by ssNMR decreases with decreasing the crystalline lamellae thickness obtained by the Raman-active longitudinal acoustic vibrational mode as well as crystallinity obtained by DSC.^35,51^ In the current study, the *T*_1C_ values of HBPEs are much shorter than those of linear PEs by Axelson et al. Therefore, the crystalline size of HBPEs is much smaller than that of linear PEs. Among the different HBPE entries, the crystalline size of entry 9 is larger than that of the other HBPEs, as a consistent *T*_1C,l_ value of around 70 s is found among the different HBPEs besides entry 9. Within one entry, the crystalline *T*_1C_ behaviors are sorted into two components, *T*_1C,l_ and *T*_1C,s_, corresponding to relatively large and small sizes of the crystals, respectively. The orthorhombic phase is composed of both long- and short-*T*_1C_ components, while the monoclinic phase, if present, is composed only of the short-*T*_1C_ component.

On the other hand, DSC experiments revealed the crystallization of entries 4, 7, 8, and 9 during cooling. However, only entry 9 exhibits a broad melting peak. The absence of melting peaks for entries 3, 4, 7, and 8 is attributed to their smaller crystalline sizes, as the *T*_1C,long_ of entry 9 is specifically 104.5 s, while those of the others are around 60–70 s. Moreover, it is difficult to develop a clear criterion to quantify the crystallinity of entry 9 based on its melting peak alone (Figure 1). Similarly, XRD revealed diffraction peaks corresponding to the orthorhombic phase in entries 3, 7, 8, and 9 (Figure 3a). However, the orthorhombic phase was not observed for entry 4, and the monoclinic phase was not detected for entries 3 and 4. Finally, we address the limitations of ssNMR spectroscopy for the determination of the crystallinity of HBPEs. The CP-*T*_1c_ relaxation curve detects double exponential components of entry 1 (Table 2), whereas saturation recovery does not capture crystal relaxation. We estimate the crystallinity of entry 1 to be <0.5% and treat entry 1 as noncrystalline. The detection limit of ssNMR spectroscopy is a crystallinity of below 0.5%. In summary, ssNMR has demonstrated advantages in characterizing crystal-related solid structures in small-sized crystals of HBPEs over conventional XRD and DSC. This is the main reason why the effect of microstructure on the solid crystalline structure of HBPEs has not been previously addressed in the literature.

### 4.2 Relationship between the Microstructure and Crystalline Structure

Here, we discuss the relationships between the branching structure, solid structure, and mechanical properties of HBPEs. First, the relationship between the branching structure and crystallinity is addressed. Entries 1, 2, 5, and 6 show noncrystalline structures, while entries 3, 4, 7, 8, and 9 show semicrystalline features. In general, a higher branching number of over 100 b/1kC leads to a completely noncrystalline HBPE. However, this is not universal. Entry 1 with a branching number of 87 b/1kC is in a noncrystalline state, whereas entry 3 with 92 b/1kC is semicrystalline. Therefore, more detailed comparisons of the microstructures are necessary. We focused on two parameters of LCB (C > 6) and SCB (C ≤ 6) numbers. The boundary line (dashed gray line) between the semicrystalline and noncrystalline states is plotted as a function of the LCB and SCB numbers of the nine HBPEs studied in this work (Figure 8). When the LCB number is less than 12 b/1kC, and SCB is less than 86 b/1kC, HBPEs are able to crystallize at ambient temperature. A larger number of either LCB or SCB leads to noncrystalline features. According to the literature, some HBPEs with a branching number of over 90 b/1kC synthesized via nickel diimine catalysis also show semicrystalline features,^3,53^ where 99% of the branches are of methyl in the work of Jian et al.^53^ Other studies reported highly branched poly­(ethylene-co-α-olefin) that shows an LCB number larger than 12 b/1kC but semicrystalline.^22,54,55^ Considering these results, we expand the noncrystalline/semicrystalline boundary from the dashed gray line to the dashed black line in Figure 8. Note that the LCBs in HBPE-co-α-olefin include those produced by the insertion of α-olefins. This diagram indicates how the molecular structures of LCB and SCB contribute to the solid structures of HBPEs.

**Figure 8:**
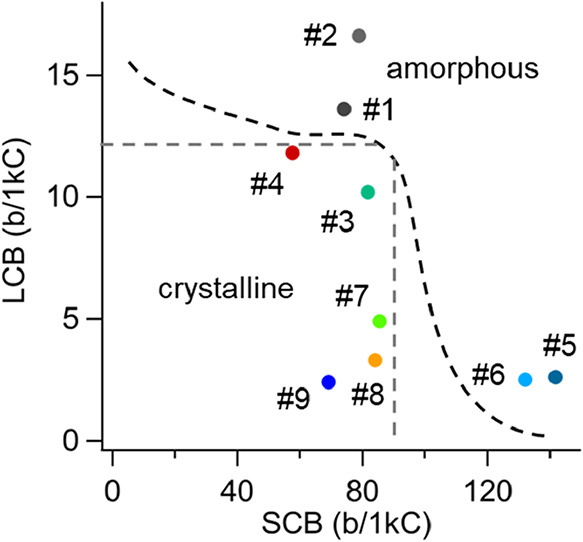
Correlation between the branching pattern and crystallizability.

Another notable result is that entries 3 and 4 possess both orthorhombic and monoclinic phases, while entries 7–9 possess only the orthorhombic phase. The former two entries have a higher LCB (over 10 b/1kC) than the latter three (below 5 b/1kC). Comparing entry 4 with entry 3, both of which have around 10 b/1kC of LCB, but entry 4 is an ethylene–octene copolymer and thus includes a 10.6 b/1kC hexyl branch structure in addition to LCB. Meanwhile, the monoclinic *T*_1C_ value (6.9 s) and crystallinity of entry 4 (1.3%) are higher than those of entry 3 (4.30 s and 0.5%). This correlation can be explained in terms of the additional hexyl branch. Such correlation is consistent with that of a previous study by Hu et al.,^25^ who reported that the monoclinic/orthorhombic ratio increases with increasing octene or butene comonomer content. Note that the methylenes in the monoclinic crystalline phase adopt parallel packing, while in the orthorhombic phase, they follow herringbone packing.^56^ Also, the crystalline packing of short-chain *n*-alkanes tends to contribute a higher chemical shift than that of PE, as revealed by Vanderhart et al.^34,57^ In the case of HBPE, though branching is attached to the polymer chain, the molecular behavior of LCB and hexyl branch may be similar to that of *n*-alkane. Therefore, the presence of the monoclinic phase is attributed to the LCB and hexyl branching content in HBPE.

### 4.3 Structure–Property Relationship

As the crystalline structure is characterized in detail, we address the structure–property relationship. Based on their difference in mechanical properties, seven HBPE entries were sorted into four categories. In the noncrystalline category I, entry 2 has a smaller *M_n_* (67 kg/mol) than that of entry 1 (163 kg/mol) and has a higher σ (0.53 MPa) and ε (2510%) than those of entry 1 (σ = 0.45 MPa, ε = 1940%). According to the literature, it was reported that PE with a higher LCB number presents a higher zero shear viscosity.^58,59^ Therefore, the slightly higher LCB number of entry 2 (16.6 b/1kC) than entry 1 (13.6 b/1kC) leads to the higher stress at break and strain at break.

Semicrystalline entries show more diverse mechanical properties than noncrystalline ones. Therefore, the diverse mechanical properties are mainly attributed to the varied crystallinities at 1.3–6.2% induced by the combination of LCB and SCB. Category II (entry 3) represents the highest *U* and highest ε values among the seven samples. These unique properties are attributed to the lowest χ_total_ of 1.3% for entry 3. Both the SCB (81.9 b/1kC) and LCB numbers (10.2 b/1kC) are close to the boundaries between the semicrystalline and noncrystalline features (Figure 9). These microstructures are the origin of the lowest χ_total_. Furthermore, the highest *M_n_* and relatively large LCB number lead to high entanglement density in entry 3. Therefore, a high entanglement density might be another source of toughness and strain at break. In category III (entries 7 and 8), all the physical properties are intermediate between categories II and IV. These properties can be explained in terms of the intermediate χ_total_ of 2.3–2.4%, which originates from a small fraction of LCB (3.3–4.9%).

**Figure 9:**
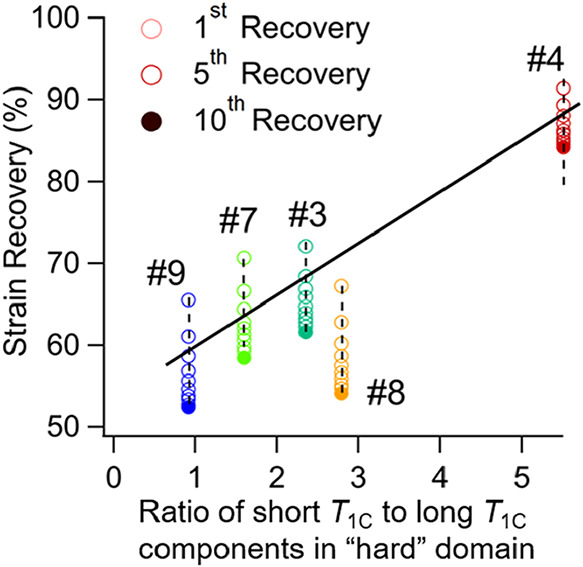
Correlation between strain recovery and the relative ratio of short-*T*_1C_ to long-*T*_1C_ components (χ_s_/χ_l_), which indicates the ratio of small crystals to relatively large crystals.

In general, high modulus and high stress at break originate from a rigid crystalline structure. In fact, entry 9 in category IV possesses the highest χ_total_ (6.2%) because of its small overall branching number (71.6 b/1kC) and the smallest LCB number (2.4 b/1kC). The *T*_1C,l_ (104.5 s) of entry 9 is also longer than the others, which indicates a larger crystalline size.^51^ Therefore, it is concluded that the higher crystallinity and larger crystalline size result in higher modulus and stress at break of the HBPEs. In entry 4, the overall branch number is very similar to that in entry 9 and much smaller than those in categories II and III. However, the LCB number (11.8 b/1kC) is much higher in entry 4 than that in entry 9 (2.4 b/1kC). Furthermore, entry 4 possesses a 10.6% hexyl branch structure. These unique microstructures lower χ_l_ (0.5%), but induce χ_l_ of 1.4% and an additional monoclinic phase (χ_m_ = 1.3%). Totally, the second-highest χ_total_ of 3.2% is introduced in entry 4. This unique arrangement of both monoclinic and orthorhombic phases might explain the second-highest modulus and stress at break for entry 4 among the seven entries.

The strain recovery of HBPEs has been reported in several previous studies. Among them, Dai et al. pointed out that a certain optimal branching microstructure of total branching and longer branches exists for good elasticity at a moderate or high molecular weight.^20^ Sun et al. proposed the importance of LCBs forming an elastic entanglement network in strain recovery.^11^ However, it is not well documented how crystalline structure contributes to strain recovery of HBPEs. Herein, we further pay attention to structural and dynamic origins for strain recovery behaviors for various semicrystalline HBPEs. Though entry 9 and entry 4 belong to the same category IV (Figure 7a), the former gives only 52%, and the latter gives 84% recovery after 10 cycles. This striking difference may be key to understanding the molecular origin of the thermoplastic elasticity of HBPEs. From the perspective of the TPE material, the ratio of the hard domain, i.e., crystallinity (χ_total_) of entry 9 (6.2%), is higher than that of entry 4 (3.2%). However, an argument based exclusively on crystallinity cannot explain the highest strain recovery of entry 4 since entries 3, 7, and 8 contribute to a lower χ_total_ of 1.3, 2.3, and 2.4%, respectively. We further examined the structure within the hard domain, which can be resolved into short-*T*_1C_ (<10 s) and long-*T*_1C_ (>10 s) components. As a result, entry 4 has the highest relative ratio of short-*T*_1C_ to long-*T*_1C_ components (χ_s_/χ_l_ listed in Table S2), as shown in Figure 9, corresponding to its unique hard domain structure dominated by small crystals. There is a good correlation between SR and χ_s_/χ_l_ for the five semicrystalline HBPEs.

To further understand the correlations between the strain recovery mechanism and crystalline structure, we conducted *T*_1C_ measurements for entries 4 and 9 after hysteresis experiments (stretched to 300% strain and back to zero stress 10 times), as they have the largest difference in strain recovery. The relaxation curves for entries 4 and 9 after the strain recovery experiment are shown in Figure S9 and Table 4. Both presented reduced *T*_1C_ and χ_l_ after stretching, which indicates the partial destruction of the relatively large crystalline structure by stretching up to 300%. The reduction in χ_l_ for entry 9 (from 3.2 to 2.1%) is much larger than that for entry 4 (from 0.5 to 0.4%), which implies that the extent of destruction in larger crystals in entry 9 is larger than that in entry 4. Conversely, the χ_s_ values of both samples increased after stretching. The χ_s_ of entry 9 increased from 3.0 to 6.3%, which is larger than the reduction in χ_l_. The monoclinic crystallinity (χ_m_) of entry 4 also increased from 1.3 to 1.9%, and the orthorhombic χ_s_ increased from 1.4 to 3.1%. These features indicate that larger crystals transform into smaller ones and some amorphous chains crystallize into smaller ones (stretch-induced crystallization). Note that in the strain recovery experiment, as shown in Figure 7b–d, there is a large strain recovery drop in the first cycle, which is the major source of the destruction of the large crystals. Therefore, although the χ_s_/χ_l_ value of one sample changes during stretching, the initial χ_s_/χ_l_ value, as plotted in Figure 9, is a good indicator to understand the strain recovery properties.

**Table 4:** *T*_1C_ Characterization Results of Entries 9 and 4 after Stretching as in the Hysteresis Experiments^a^

entry	phase	*T*_1C,l_/s	*f*_l_ (%)	*T*_1C,s_/s	*T*_1C,a_/s	*f*_a_ (%)	χ_l_/%	χ_s_/%
9	O	95.8 ± 7.4	25	6.98 ± 1.32	0.42 ± 0.02	50	2.1	6.3
4	O	56.2 ± 12.7	8.2	1.68 ± 1.41	0.30 ± 0.02	69	0.4	3.1
	M			6.01 ± 0.50	0.35 ± 0.02	80		1.9

a The same format is used as in Table 2.

The above ssNMR analysis implies that the key structure for the thermoplastic elasticity of HBPE is the small hard domain. The following molecular mechanisms have been proposed to explain the thermoplastic elasticity of HBPEs. Scheme 1a shows the molecular image of HBPE with low strain recovery, where the hard domain is composed of both large and small crystals, like entry 9 in this work. Scheme 1b shows the molecular image of HBPE with high strain recovery, where the hard domain is composed of predominantly small crystals, like entry 4. While stretching, as shown in Scheme 1c, the HBPE molecules align with the stretching direction. During alignment, part of the large crystals experiences concentrated stress and is broken down into small crystals, and some polymer chains crystallize into the small crystals, as highlighted in red. The remaining large anisotropic crystals are physically connected to the small ones via tie molecules. These chain networks do not allow large anisotropic crystals to fully relax to their original orientations and locations, as shown in Scheme 1e. Meanwhile, the small crystals can be aligned with the stretching direction without destroying the original ones, as shown in Scheme 1d (as proven by the ^13^C *T*_1C_ results listed in Table 4). Also, stretching induces the formation of new small crystals. However, the number of new crystals formed is limited. Therefore, the predominantly small crystals relax toward their original orientations and locations. Therefore, HBPEs with small crystals exhibit better strain recovery than the HBPEs with large crystals. These dynamic microscopic crystalline structures are the origin of the stress-recovery properties of HBPEs. Combined with the correlation between the branching structure and crystalline structure, the thermoelastic properties can guide the design of the microstructures of HBPE. A high stress recovery may be realized by designing an HBPE molecular structure that prefers small-size crystals or by using certain preparation methods that can prevent large-size ones in the original HBPE material.

**Scheme 1:**
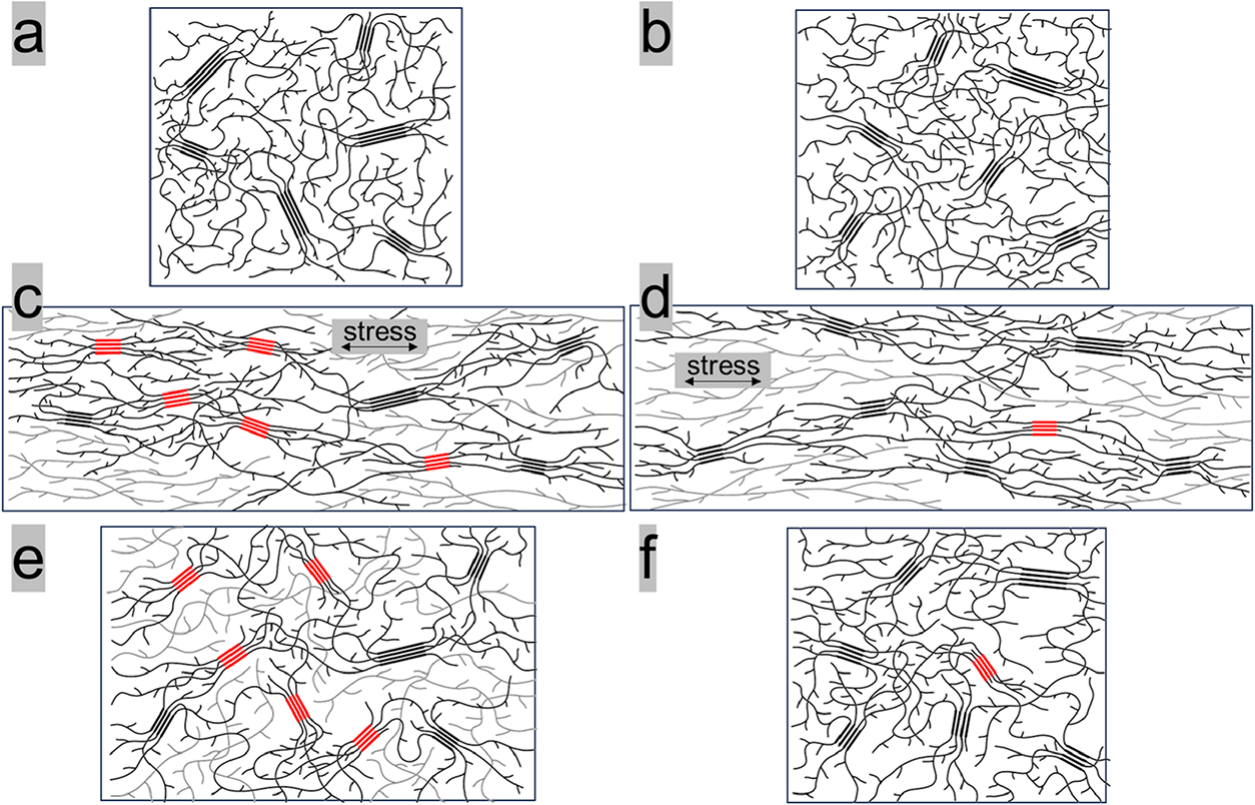
Illustrations of HBPE Molecules, Where the Bold and Parallel Black Lines Correspond to Crystallized Stems: (a) HBPE That Has Both Large and Small Crystals; (c) During Stretching, Where Part of the Large Crystals Are Destroyed and New Small Crystals Are Formed, Highlighted in Red; (e) after Hysteresis. (b) HBPE That Has Fewer Large Crystals and More Small Crystals; (d) During Stretching; (f) after the Hysteresis Process

## 5 Conclusions

In this work, we investigated the impact of microstructures on the solid structure as well as the mechanical properties of various HBPEs synthesized via nickel diimine catalysts. Through the CP-*T*_1C_ relaxation method in ssNMR, orthorhombic and monoclinic crystalline phases were confirmed for HBPEs with branching numbers in the range of 70–95 b/1kC. The interplay between the branching number and branching pattern in determining the crystalline structure and their size in HBPEs from chain-walking polymerization was quantified. Only HBPEs with both LCB (C > 6) number lower than 12 b/1kC and SCB (C ≤ 6) number lower than 85 b/1kC were semicrystalline. The *T*_1C_ behaviors can be sorted into long and short components in the orthorhombic phase, where the former ranges from 64 to 104 s and the latter ranges from 3.3 to 7.1 s. The long and short *T*_1c_ values are associated with crystal size. The monoclinic phase showed short *T*_1C_ values of 4.3–6.9 s. The crystallinities (χ_total_ = 1.3–6.2%) of the orthorhombic and monoclinic phases, including the fraction of each *T*_1C_ component, were successfully determined by the relaxation methods. Furthermore, the stress–strain experiments classify HBPEs into four different categories. One is associated with (i) noncrystalline HBPE, and three are associated with semicrystalline HBPEs with (ii) high and (iii) intermediate toughness, and (iv) high modulus/high stress at break. The high and intermediate toughness properties are attributed to the crystallinity differences associated with the LCB fraction and molecular weight differences. The high mechanical properties of HBPEs originate from their higher crystallinity (6.2 and 3.0%). However, the highest crystallinity sample exhibits the worst strain recovery, while the second-highest crystallinity sample exhibits the best strain recovery among the five semicrystalline HBPEs. The controversial strain recovery behaviors were interpreted in terms of the distribution of different crystal sizes. The second-highest crystallinity with small crystals results in excellent stress–strain recovery, while the highest crystallinity with large crystals results in the worst strain recovery. A detailed structure–property relationship in HBPEs will be beneficial to understand the molecular origin of the thermoplastic elasticity of HBPEs and guide the design of unique thermoplastic elastomers of HBPEs by controlling the branching structures and further controlling their solid-phase structures.

## Supplementary Material



## Notes

Supporting Information experimental results and synthesis of the HDPE sample (PDF)
