# Supplementary material for: Molecular Origins of Thermoplastic Elasticity of Highly Branched Polyethylene as Revealed by Solid-State NMR Spectroscopy
Source: Macromolecules. 2026 Jul 17;59(14):8497–508. doi: 10.1021/acs.macromol.6c01367 (PMC13421960; doi:10.1021/acs.macromol.6c01367)
Supplement: Supplementary file 1 [file ma6c01367_si_001.pdf]

SUPPORTING INFORMATION

Molecular Origins for Thermoplastic Elasticity of Highly Branched Polyethylene as Revealed by Solid-state NMR Spectroscopy

Bohao Peng, Keaton M. Turney, Walter G. Romano, James M. Eagan* and Toshikazu Miyoshi*

School of Polymer Science and Polymer Engineering, The University of Akron, Akron, Ohio 44325-3909, United States.

*Corresponding Author email: eagan@uakron.edu, miyoshi@uakron.edu

1. Supplementary Figures and Tables.

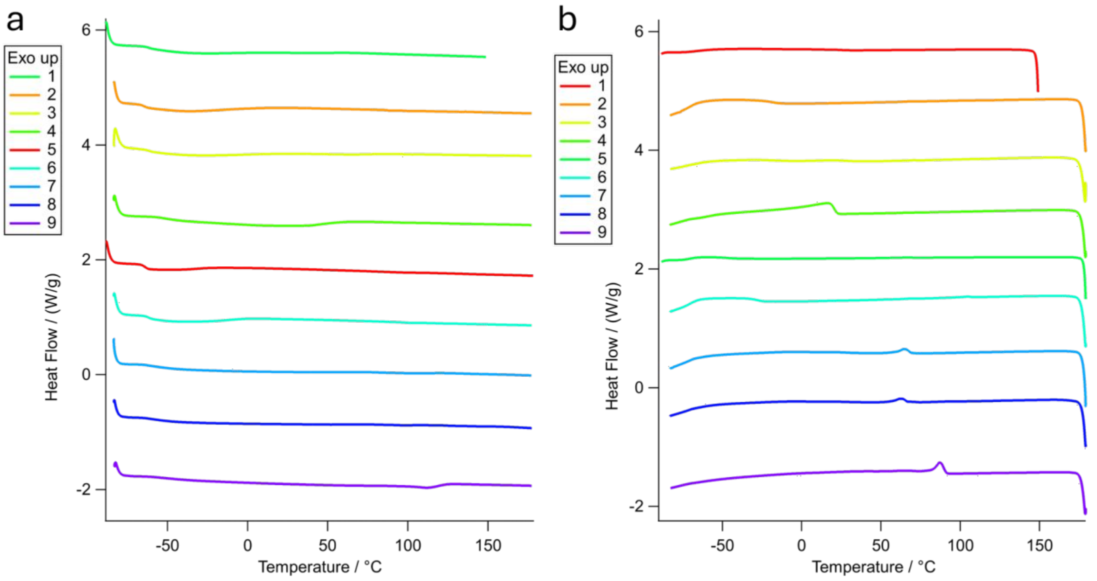

Figure S1: DSC second (a) heating and (b) coolingcurves of all HBPE entries at the cooling/heating rate of 10 °C/min. Curves are shifted in benefit for presentation. Left axis labels for heat flow are shown only to present the scale of melting/crystallization peaks if observed.

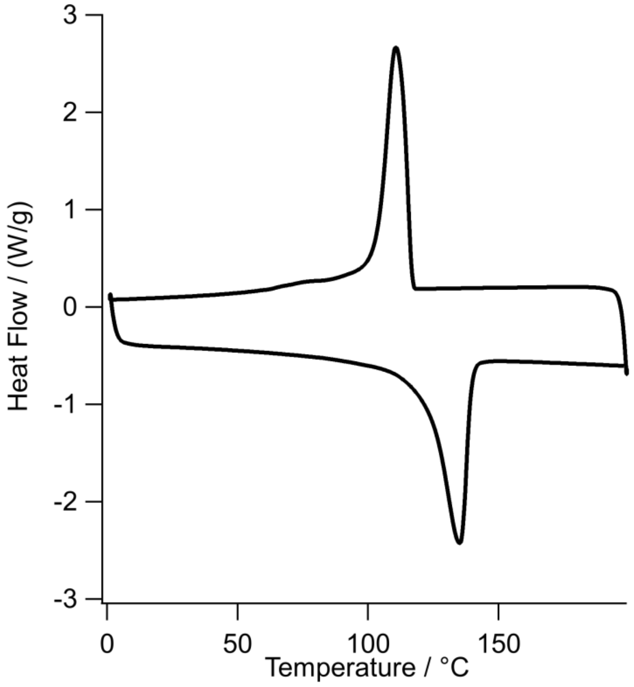

Figure S2: DSC thermograms of HDPE sample at the rate of 10 °C/min.

Table S1: T_{1C} of the amorphous peaks (/ ms)

Chemical Shift/ppm	37.9	35.1	33.6	30.5	27.9	20.4
Assignment	αB_1	1,4-$\alpha' B_1$	brB_1	main	βB_1	$1B_1$
1	179.6		314.6	245.3	192.4	441.7
2	175.7		239.9	254.6	202.4	533.5
3	177.8		293.5	278.6	198.3	527.2
4	206.5		500.8	293.1	207.3	552.1
5	167.8	169.3	259.4	254.5	202.0	453.2
6	175.2	169.4	267.6	281.6	210.1	502.7
7	206.2			300.1	212.1	541.1
8	189.5			284.3	209.0	509.1
9	202.9			369.0	262.1	618.9

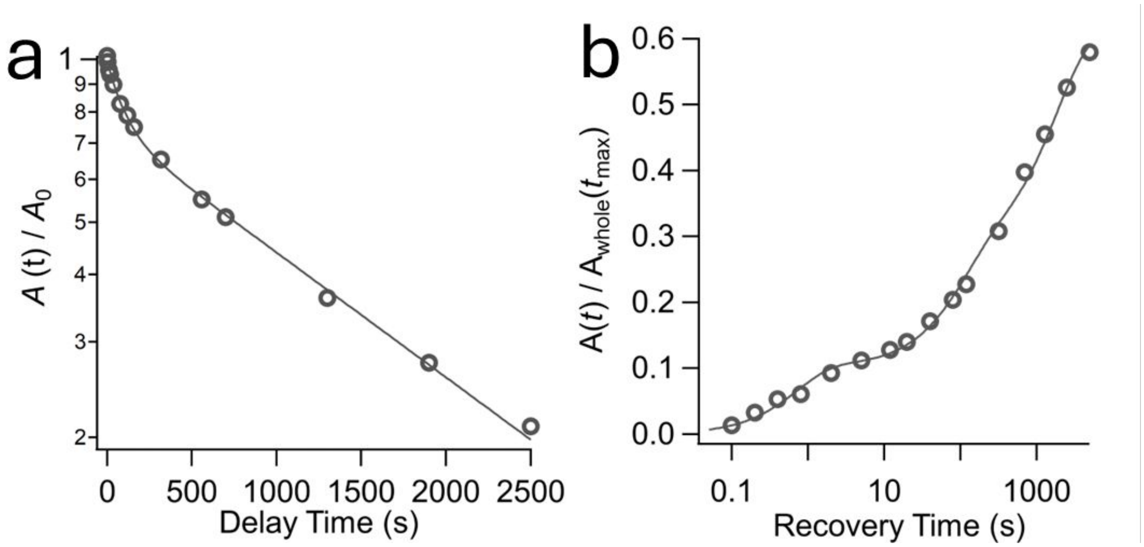

Figure S3: Full data plot of CP- T_{1C} integration area plot and fitting curves of the HDPE sample. Full plot of integration area analysis from 33.9 to 31.9 ppm from SR/T1C experiments of the HDPE sample.

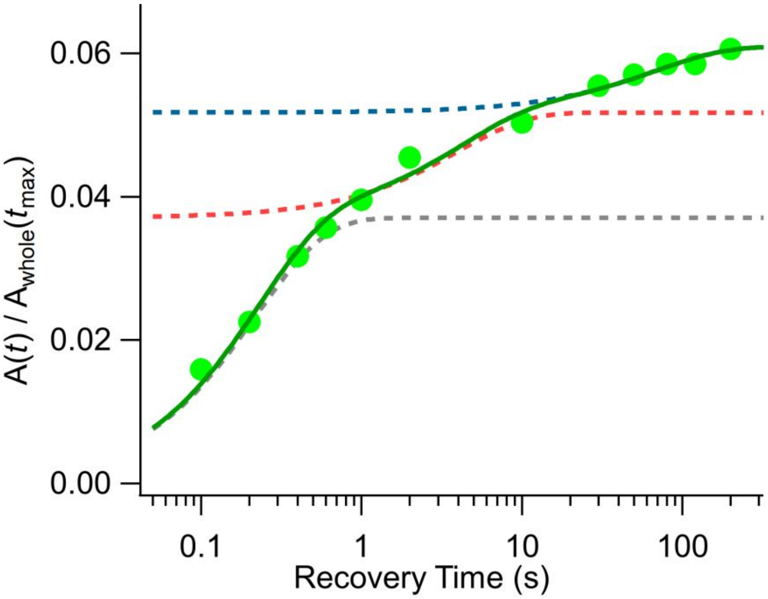

Figure S4: Signal recovery in the SR-T_{1C} experiments of HBPE entry 7.

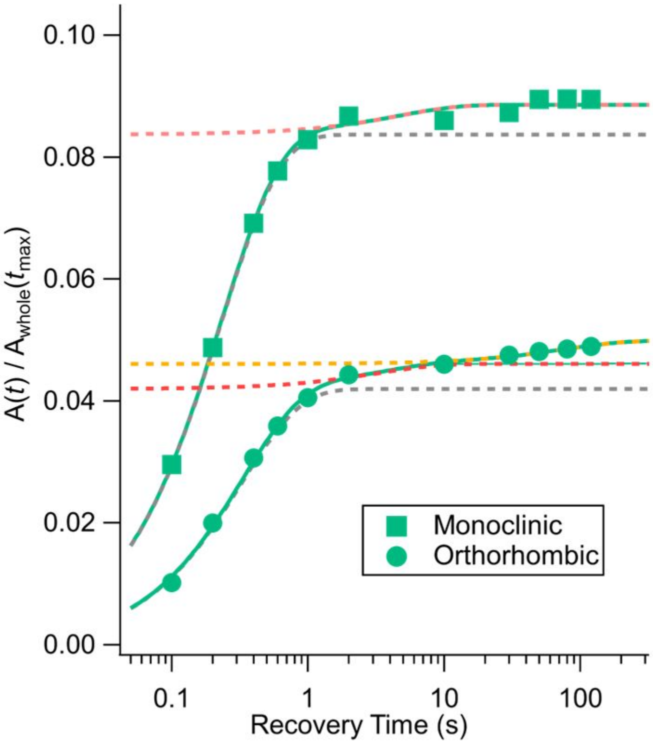

Figure S5: Signal recovery in the SR-T_{1C} experiments of HBPE entry 3.

Table S2: Best fit parameters of SR signal recovery analysis.

Entry	Phase	$T_{1C,a}'$ / s	fa'	fs'	fl'	A (t_{max}) / A_{whole}(t_{max})	χ_s/χ_l
HDPE	O	0.72 ± 0.15	0.166 ± 0.009	0.282	0.552 ± 0.018	0.611	0.51
9	O	0.34 ± 0.02	0.462 ± 0.012	0.257	0.281 ± 0.014	0.115	0.91
8	O	0.25 ± 0.01	0.643 ± 0.015	0.263	0.094 ± 0.023	0.136	2.86
7	O	0.22 ± 0.02	0.608 ± 0.023	0.241	0.151 ± 0.033	0.134	1.67
3	O	0.33 ± 0.01	0.840 ± 0.018	0.083	0.077 ± 0.022	0.050	2.27
	M	0.23 ± 0.01	0.945 ± 0.014	0.055	–	0.089	
4	O	0.15 ± 0.02	0.753 ± 0.042	0.177	0.070 ± 0.024	0.070	5.56
	M	0.14 ± 0.01	0.842 ± 0.023	0.158	–	0.080	
9 (stretched)	O	0.28 ± 0.03	0.472 ± 0.020	0.355	0.173 ± 0.026	0.119	2.04
4 (stretched)	O	0.08 ± 0.01	0.580 ± 0.029	0.363	0.057± 0.046	0.075	12.5
	M	0.10 ± 0.01	0.771 ± 0.024	0.229	–	0.084	

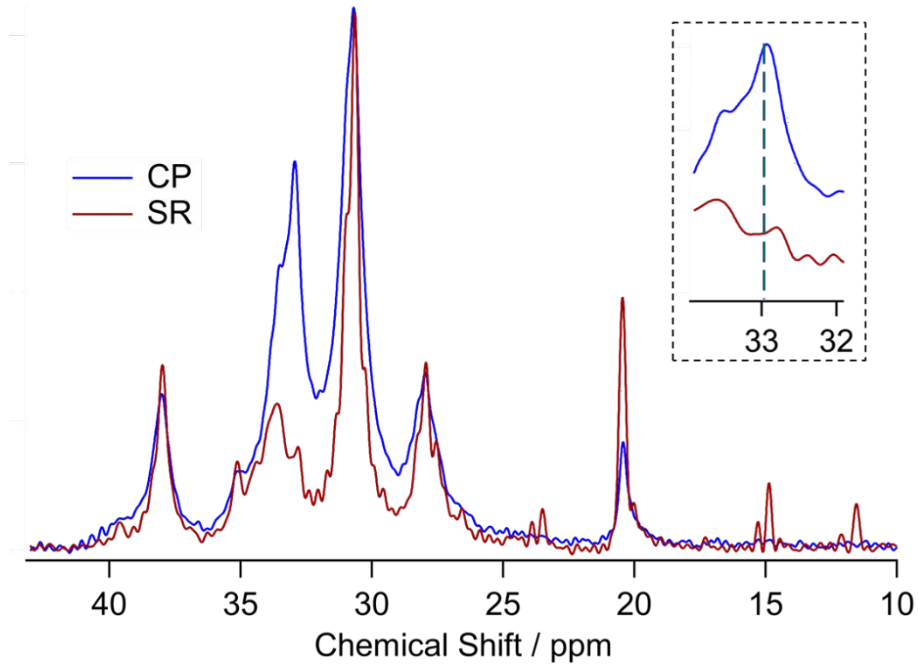

Figure S6: Comparison of CP-MAS spectrum and SR-T_{1C} spectrum at the recovery time of 300 s of entry 8. Notably, one SR spectrum behavior that could be highly misleading is that there is a peak at 32.8 ppm that is actually not crystalline peak, especially for lower crystalline entries 4, 3, 7 and 8. In the SR spectrum, the apparent peak at 32.8 ppm also consists of a methylene near LCB end (3Bn, n>5) signal in addition to the orthorhombic crystalline peak.

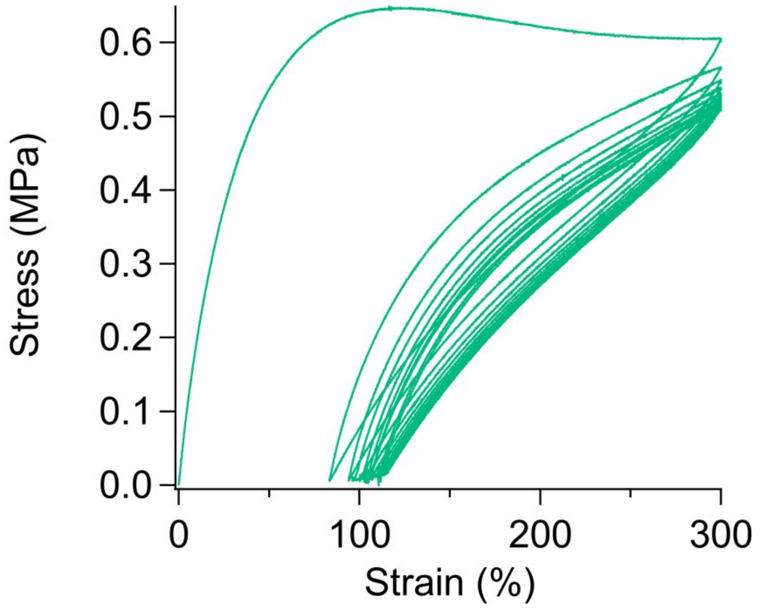

Figure S7: Stress strain curves from hysteresis experiments with ten cycles up to 300% strain for entry 3.

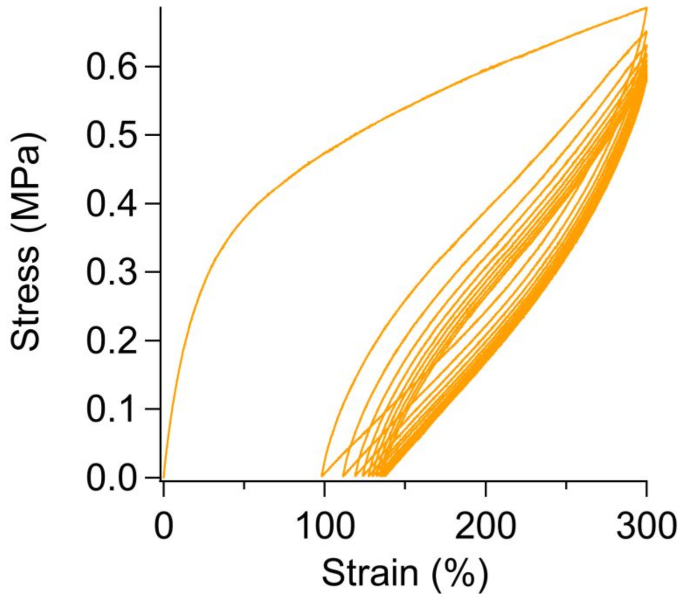

Figure S8: Stress strain curves from hysteresis experiments with ten cycles up to 300% strain for entry 8.

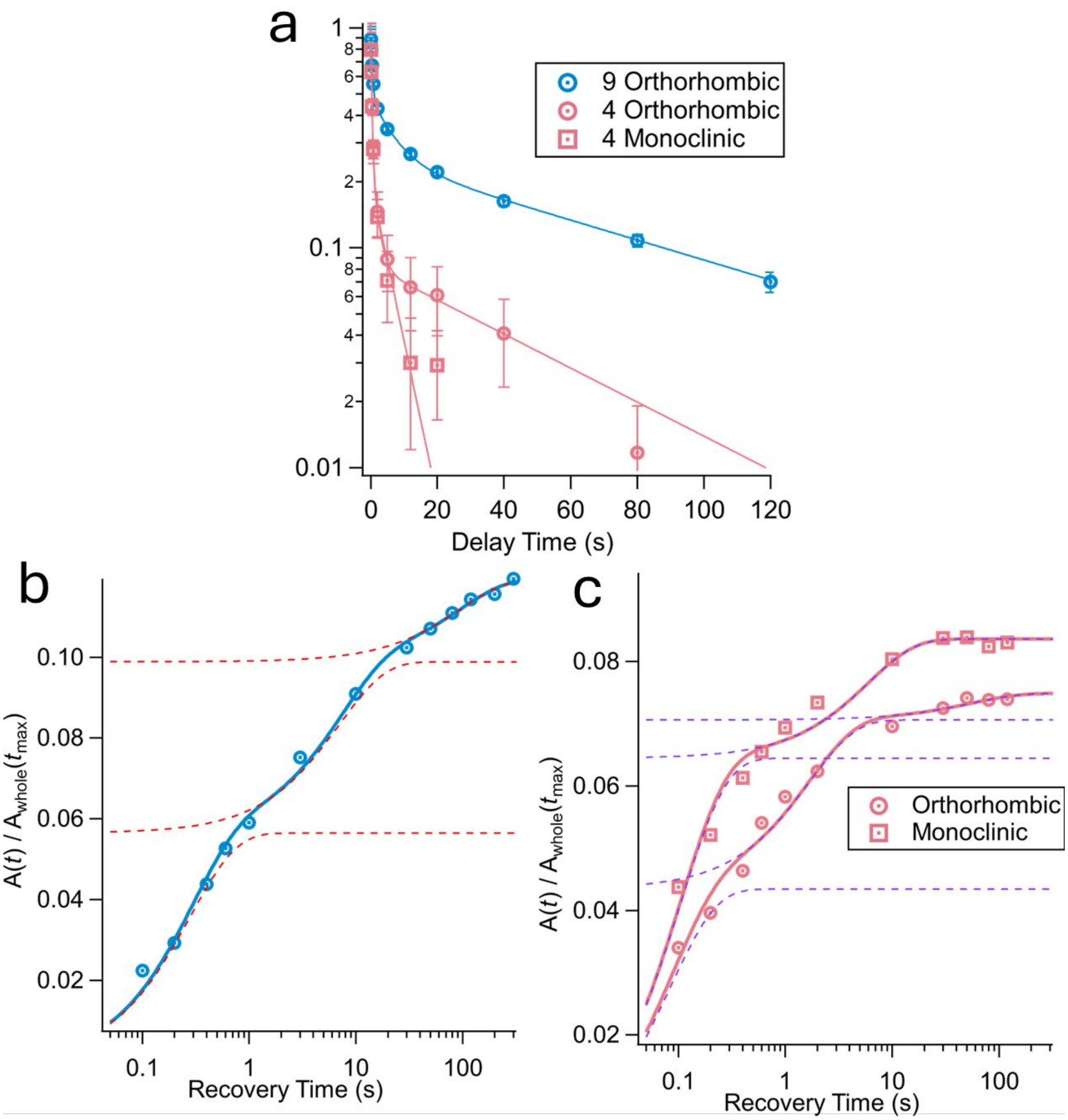

Figure S9. ssNMR characterizations of HBPE entries 9 and 4 after stretching as in hysteresis experiment: (a) CP-T_{1C} relaxation behaviors. (b) Signal recovery in SR-T_{1C} experiments of entry 9 after stretching. (c) Signal recovery in SR-T_{1C} experiments of entry 4 after stretching.

2. Synthesis of Polymer samples

The HDPE sample is synthesized through Ziegler-Natta catalyst with molecular

characteristics as shown in the following table.

Table S3: Molecular characteristics of HDPE sample.

T_m [°C]	T_c [°C]	ΔH°_{fus} [J/g]	Crystallinity* (by DSC)	M_n [g/mol]	*Đ*
136.5	110.7	192.2	65.6%	189,000	4.1

*Crystallinity % was calculated by comparing heat of fusion to 100% crystalline heat of fusion (293 J/g).

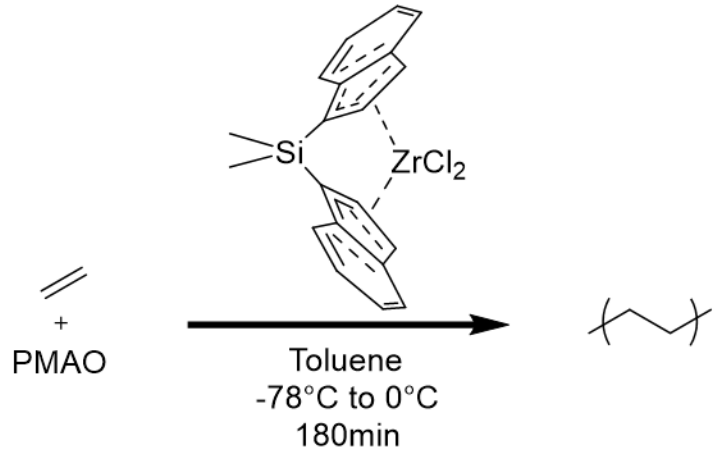

Synthesis. A Fischer-Porter Vessel with a stir bar was flame dried and transferred into a N_2 Atmosphere Vigor Sci-Lab glovebox with a Swagelok pressure head. Once inside, 50mL of dry Toluene was added along with 1.04g of PolyMethylAluminOxane (PMAO). The vessel was then sealed and removed from the glovebox and moved to a -78°C bath of dry ice + acetone to cool. After 20 minutes of cooling, the weight of the vessel was recorded. The vessel was then evacuated using a Schlenk line until no foaming of the solution was apparent. The evacuated vessel was then cooled again for 10-15 min using the same cold bath. Once cooled, the empty evacuated vessel was again weighed and cooled again to -78°C. The ethylene tank was then turned on and a charge of gas was injected into the fisher porter reaction and weighed to be 1.05g of ethylene gas. The vessel was kept cold while in the glovebox, 9 mg of Rac-Dimethylsilylbis(1-indenyl)zirconium dichloride (SiZr) catalyst was weighed along with 1.04 mg of PMAO and loaded in a syringe and needle stopped by a septum. This was removed from the glovebox and set near the cold vessel. This catalyst solution was injected into the vessel while it was cooled to -78°C. Once injected, the vessel

was sealed and moved to an ice-water bath at 0°C and stirred for 3 hours. At the end of the allotted time, the reaction was quenched with 5% HCl in MeOH (25ml) and stirred for 60 min to dissolve the aluminum salts. The polymer was precipitated in excess MeOH, decanted, filtered, washed with more MeOH, and dried in vacuum at 60°C overnight.

Materials.
Ethylene (Polymer Grade 99.9%) was purchased from Matheson Gas. PMAO-IP (13wt% Al in toluene) was purchased from Nouryon Functional Chemicals LLC and used as received. SiZr catalyst was purchased from Strem Chemicals Incorporated and used as received. Toluene (≥99.5%) was purchased from Millipore Sigma and purified by passing through a Vigor Tech Solvent purification system. Hydrochloric acid (Certified ACS Plus) and Methanol (Certified ACS) were purchased from Fisher Chemical and used as received.

Reference

(1) Peng, B.; Turney, K.; Eagan, J.; Miyoshi, T. Branching Pattern, Distribution, and Chain-Walking Mechanism of Highly Branched Polyethylenes Synthesized by α-Diimine Ni(II) Catalysts as Studied via 13C Solution-State NMR Spectroscopy. *Macromolecules* **2025**, *58* (15), 7871–7883. DOI: 10.1021/acs.macromol.5c01063.
